# Filtering Characteristics of Phonon Polaritons Waves Based on Dielectric-h-BN-Dielectric Structure in Mid-Infrared Band

**DOI:** 10.3390/nano10050878

**Published:** 2020-05-01

**Authors:** Ming Cai, Shulong Wang, Zhihong Liu, Yindi Wang, Tao Han, Hongxia Liu

**Affiliations:** Key Laboratory for Wide Band Gap Semiconductor Materials and Devices of Education, School of Microelectronics, Xidian University, Xi’an 710071, China; cm9999787@163.com (M.C.); zhliu@xidian.edu.cn (Z.L.); wangyindi4213@126.com (Y.W.); 15639119745@163.com (T.H.)

**Keywords:** phonon plaritons, filtering characteristics, h-BN, mid-infrared band

## Abstract

Hyperbolic materials can be used to excite hyperbolic phonon polaritons in specific frequency bands, which causes abrupt interfaces with fluctuations of permittivity and different transmission characteristics at different incident wavelengths. Using the quasi-static approximation, the filtering characteristics of hexagonal Boron nitride (h-BN) and the transmission characteristics of phonon polaritons waves on a dielectric-h-BN-dielectric structure were studied in the paper. The results show that a smaller relative permittivity of the materials above and below h-BN and a thicker h-BN (ε_1_ = 1 (air), ε_2_ = 3.9 (SiO_2_), d = 100 nm) will lead to better filtering characteristics for different wavenumbers’ incident waves (propagation length from 0.0028 μm to 1.9756 μm). Simulation results in COMSOL validated the previous theoretical calculations. Moreover, the transmissivity and 3dB bandwidth of the type-II band were calculated with different structure widths. The maximum transmissivity of ~99% appears at a width of 100 nm, and the minimum 3dB bandwidth reaches 86.35 cm^−1^ at a structure width of 1300 nm. When the structure width meets or exceeds 1700 nm, the 3dB bandwidth is equal to 0, and its structure length is the limit for the filter application. These characteristics reveal the excellent filtering characteristics of the dielectric-h-BN-dielectric structure, and reveal the great potential of using the dielectric-h-BN-dielectric structure to design optical filter devices with excellent performance in mid-infrared bands.

## 1. Introduction

Polaritons [1,2,3], the collectively excited state produced by the coupling of photons and matter, have a unique potential to create new applications beyond traditional electronics and photonics [4]. The phonon polaritons [5,6] are the collective oscillations resulting from the coupling between photons and optical phonons [7,8] in polar dielectrics, whose main frequencies are in the range of mid-IR wavelengths. When the oscillation frequency reaches the photon polaritons’ resonance frequency [9], the relative permittivity of the polar materials will undergo a huge increase—even increasing from a negative value or zero to a large positive value [10,11]. The huge change of the relative permittivity under different incident wavenumbers will cause a huge change in the transmission length.

In addition, hyperbolic materials [12,13] have attracted many attentions for the applications in optoelectronics such as vibrational spectroscopy and stimulated Raman scattering. Hexagonal Boron nitride (h-BN) [14] is a natural hyperbolic material with a wide bandgap of about 6 eV [15]. h-BN has an excellent material property—that is, the relative permittivity with opposite signs for in-plane and out-plane in the mid-infrared band. Applying this special property, the hyperbolic phonon polaritons (HPPs) can be excited, which causes particularly strong phonon resonances in the mid-infrared band. In this case, the strong phonon resonance characteristics of the HPPs in h-BN can be used to design high-performance modulators, sensors and other devices. Combining the tunable photoelectric materials [16,17,18], some researchers designed a VO_2_-h-BN-graphene asymmetric transmission structure [19] and investigated the high-efficiency modulation characteristics of the coupling between different polaritons in graphene/h-BN heterostructures for modulator [20,21] and sensor [20] applications. These works have made significant progress, and proved the potential of h-BN to be applied in optical devices. Until now, however, very few reports have been focused on the investigation of the propagation characteristics to analyze the filtering characteristics of HPPs in h-BN, which is extremely important for the optical device designs. 

In order to design nano-optical filter devices with a high performance based on the phonon polaritons in h-BN, the propagation and filtering characteristics of h-BN have been studied in this paper. After giving an introduction about the background and motivation of the work, Section 2 will explain the approach of the theoretically analytical calculation. Section 3 will provide the analytical calculation results and the verification using numerical simulations with the assistance of the COMSOL tool. Different materials above and below h-BN and different thicknesses of h-BN are investigated via simulations. In the dielectric-h-BN-dielectric structure, at different incident wavenumbers, the propagation lengths [22] are different, which can be used to realize the filtering function. By optimizing the structure width, an improved performance of the structure can be achieved. The final results show the excellent filtering characteristics of the dielectric-h-BN-dielectric structure, which can be of great interest for filter applications in the mid-infrared range.

## 2. Structural Design and Methodology

**Figure 1:**
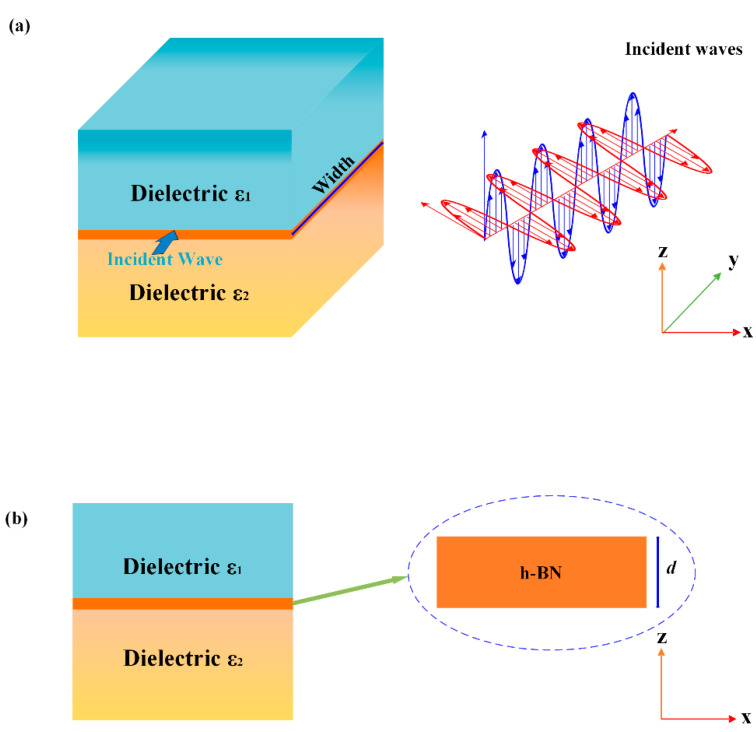
Stereograph and sectional schematics of the structures: (**a**) 3D layout waveguide structure; and (**b**) cross-section structure.

**Figure 2:**
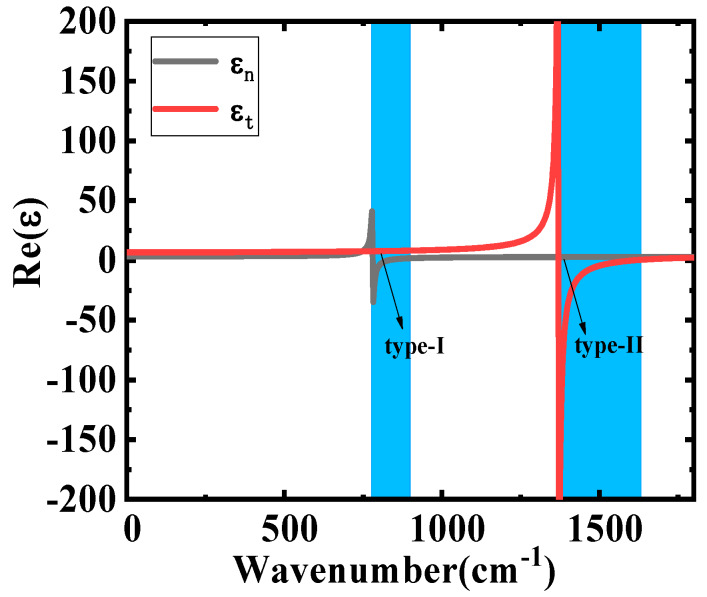
Re(ε) as a function of wavenumber.

Figure 1 shows the three-dimensional and cross-sectional model of the structure. The structure is composed of sandwiched dielectric-hexagonal Boron nitride (h-BN)-dielectric layers. In this paper, air is used as the material above the h-BN, while Si or SiO_2_ is used as the material below the h-BN. The SiO_2_ here means amorphous silica. The incident waves are along the y direction, and the dimensions of the dielectric layers are semi-infinite in the z direction. Here, parallel light beams are used as the incident waves.

h-BN possesses many excellent optical properties, such as its permittivity, which can be tuned by different incident wavenumbers. h-BN has two kinds of active phonon modes, which correspond to two frequency bands with hyperbolic characteristics in the mid-infrared band. One is the normal plane phonon mode, with a ω_TO_ value of 780 cm^−1^ and a ω_LO_ value of 830 cm^−1^, while the other is the basal plane phonon mode, with a ω_TO_ value of 1370 cm^−1^ and a ω_LO_ value of 1610 cm^−1^ [15]. The lower frequency normal plane phonon mode corresponds to the type-I band (ε_n_ < 0 and ε_t_ > 0, ε_n_ and ε_t_ are the tangential relative permittivity and the normal relative permittivity of h-BN, respectively). The higher frequency basal plane phonon mode corresponds to the type-II band (ε_n_ > 0 and ε_t_ < 0) [23]. The Reststrahlen (RS) bands are categorized as RS-I and RS-II regions, corresponding to type-I and type-II bands in h-BN [19]. The relationship between the wavenumber (ω) and the relative permittivity (*ε*) of the h-BN in RS bands is given by [24]

(1) $$\varepsilon_{n(t)}=\varepsilon_{\infty ,n(t)}+\varepsilon_{\infty ,n(t)}\times \frac{{\left(\omega_{LO,n(t)}\right)}^{2}-{\left(\omega_{TO,n(t)}\right)}^{2}}{{\left(\omega_{TO,n(t)}\right)}^{2}-\omega^{2}-i\omega \Gamma_{n(t)}}$$

where *ε_∞,n_* denotes the normal high-frequency limited relative permittivity, and its value is 2.95. *ε_∞,t_* refers to the tangential high-frequency limited relative permittivity, whose value is 4.87. Γ_n_ and Γ_t_ denote the normal optical phonon bandwidth and the tangential optical phonon bandwidth, respectively, with values of 4 cm^−1^ and 5 cm^−1^ [15,20]. Figure 2 shows the real part of the relative permittivity (Re(ε)) as a function of the wavenumber.

As shown in Figure 2, h-BN has two RS bands in opposite symbolic real parts of the normal relative permittivity and the tangential relative permittivity. The first RS band appears at about 800 cm^−1^ wavenumbers (corresponding to the type-I band), while the second RS band appears at about 1400 cm^−1^ wavenumbers (corresponding to the type-II band).

With the introduction of quasi-static approximation, the relationship between wave vectors β_h-BN_ and the relative permittivity can be given by [24,25,26]

(2) $$\beta_{h-BN}=-\frac{\Psi }{d}\{\tan^{-1}[\frac{\varepsilon_{1}}{\varepsilon_{t}\Psi }]+\tan^{-1}[\frac{\varepsilon_{2}}{\varepsilon_{t}\Psi }]+n\pi \}$$

where ε_1_ and ε_2_ stand for the materials’ relative permittivity above and below the h-BN. d represents the thickness of the h-BN. Ψ is given by (ε_n_/ε_t_)^1/2^/*i*. *n* refers to the excitation order number of the different phonon polaritons waves (n = 0,1,2…). According to the electromagnetic transmission theory, the device transmissivity T can be given by

(3) T=12Re(Et×Ht*)12Re(Ei×Hi*)

where E_t_ and H_t_ stand for the transmitted electric and the magnetic field intensity, respectively. E_i_ and H_i_ represent the incident electric and the magnetic field intensity, respectively.

## 3. Results and Discussion

**Figure 3:**
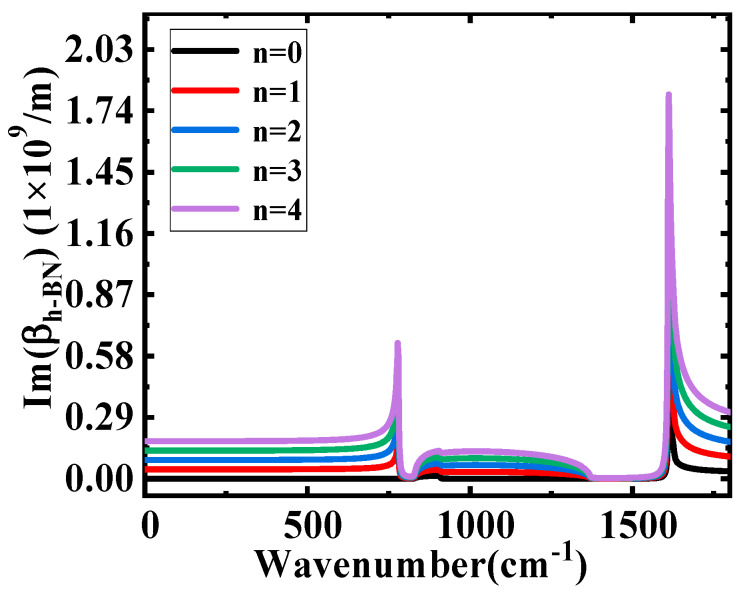
Im(β_h-BN_) as a function of wavenumber.

Figure 3 shows the change of the transverse wave vector’s imaginary part (Im(β_h-BN_)), with the increasing wavenumber in different excitation mode n. In order to make comparison easier, the next analysis is only focused on the mode n = 1. According to Equations (1) and (2), the Im(β_h-BN_) can be influenced by the thickness of h-BN, the materials above and below the h-BN and the wavelength of the incident waves.

**Figure 4:**
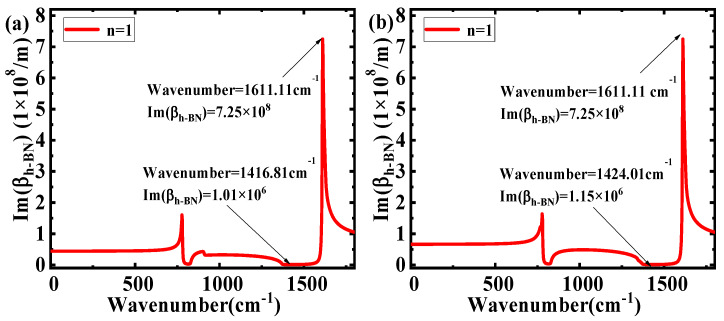
Im(β_h-BN_) as a function of wavenumber in different ε_1_ and ε_2_: (**a**) ε_1_ = 1 (air), ε_2_ = 3.9 (SiO_2_), d = 50 nm; and (**b**) ε_1_ = 1 (air), ε_2_ = 11.9 (Si), d= 50 nm.

**Figure 5:**
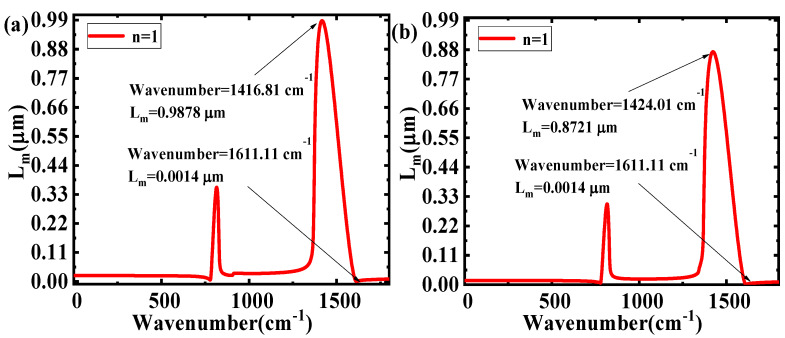
L_m_ as a function of wavenumber in different ε_1_ and ε_2_: (**a**) ε_1_ = 1, ε_2_ = 3.9, d = 50 nm; and (**b**) ε_1_ = 1, ε_2_ = 11.9, d = 50 nm.

The plots of Im(β_h-BN_) on the wavenumbers of different ε_2_ are shown in Figure 4. Comparing Figure 4a,b, it can be seen that as the wavenumber increases, the range of Im(β_h-BN_) has an obvious decrease (1.01 × 10^6^–7.25 × 10^8^ to 1.15.06 × 10^6^–7.25 × 10^8^). The results illustrate that a larger ε_2_ will cause a larger range of Im(β_h-BN_). Applying the expression of propagation length L_m_ = 1/Im(β_h-BN_), the relationship between the wavenumber and L_m_ at different ε_2_ is shown in Figure 5. The range of L_m_ obviously decreases (from 0.0014–0.9878 μm to 0.0014–0.8721 μm) with the increasing ε_2_. The main reason for this is that materials with higher dielectric permittivity values above and below h-BN directly increase the light energy loss in h-BN, which will cause a shorter L_m_. Combining the theory of electromagnetic field, a larger range of L_m_ in different wavenumbers show the better selective properties and the excellent filtering characteristics. Moreover, according to Equation (2), the effect of ε_1_ on the filter characteristics is the same as that of ε_2_.

**Figure 6:**
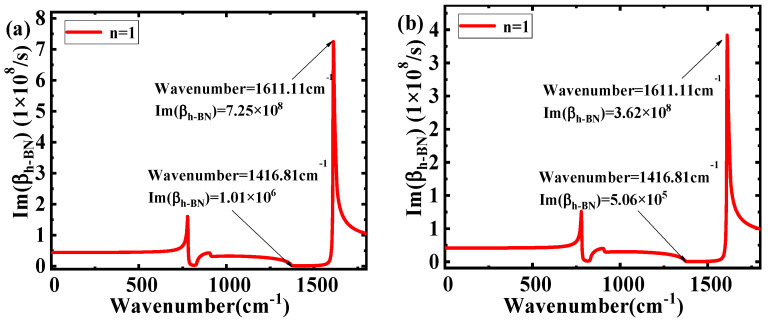
Im(β_h-BN_) as a function of wavenumber in different thicknesses of h-BN: (**a**) ε_1_ = 1, ε_2_ = 3.9, d = 50 nm; and (**b**) ε_1_ = 1, ε_2_ = 3.9, d = 100 nm.

**Figure 7:**
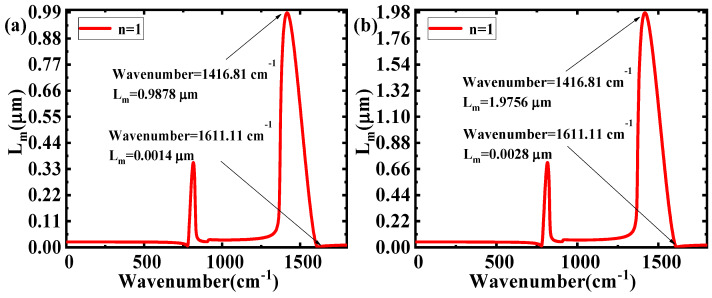
L_m_ as a function of wavenumber in different thicknesses of h-BN: (**a**) ε_1_ = 1, ε_2_ = 3.9, d = 50 nm; and (**b**) ε_1_ = 1, ε_2_ = 3.9, d = 100 nm.

**Figure 8:**
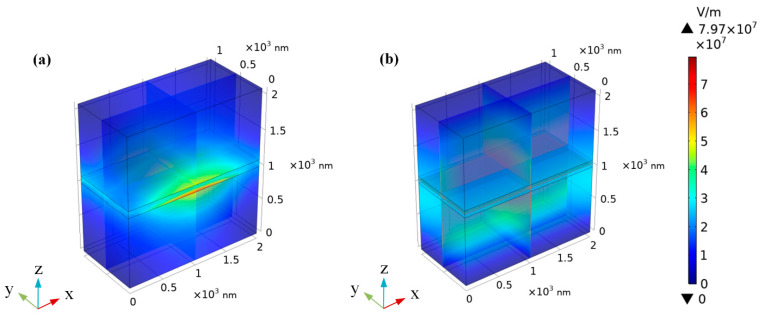

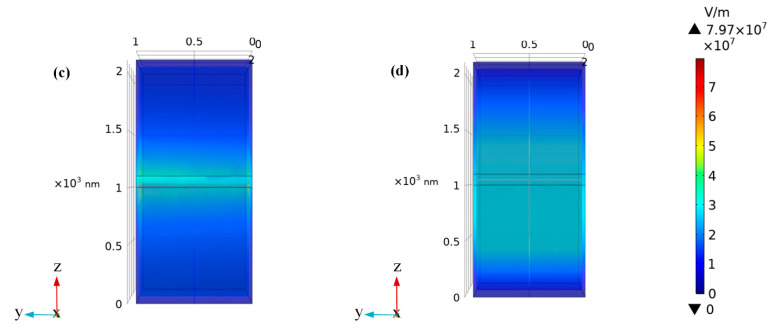
Electric field distribution of the structure in 1000 nm structure width: 3D layout waveguide structure in the (**a**) 1416.81 cm^−1^ and (**b**) 1611.11 cm^−1^ wavenumber; and cross-section of the yz plane in the (**c**) 1416.81 cm^−1^ and (**d**) 1611.11 cm^−1^ wavenumber.

Figure 6 shows the relationship between Im(β_h-BN_) and the wavenumber for different d. With the increase of d, the range of Im(β_h-BN_) has an obvious decrease (from 1.01 × 10^6^–7.25 × 10^8^ to 5.06 × 10^5^–3.62 × 10^8^). According to the equation L_m_ = 1/Im(β_h-BN_), the relationship between the wavenumber and L_m_ for different d is shown in Figure 7. The range of L_m_ increases hugely (from 0.0014 μm~0.9878 μm to 0.0028–1.9756 μm), with d changed from 50 nm to 100 nm. The increase of d in h-BN reduces the light energy loss, corresponding to the increase of L_m_, and is the main reason for the findings. As a larger range of L_m_ at different wavenumbers means better selectivity, the results show that the filtering characteristics can be vastly improved by increasing the d. From the above discussions, the larger ε_2_ will reduce the filtering characteristic for incident waves in different wavenumbers. When increasing the thickness of h-BN, the filtering characteristic can be improved for different wavenumbers’ incident waves. In order to verify the above-mentioned theoretical analysis, the finite element method (FEM) package in the RF module of COMSOL Multiphysics 5.4 (COMSOL Inc., Stockholm, Sweden) was adopted to simulate the electric field distribution. The mode analysis with a scattering boundary was used under open boundary condition. In the simulation, the whole device size was set as 2000 nm × 1000 nm × 2100 nm in x, y and z directions, respectively. The dielectric materials above and below h-BN are air and SiO_2_, respectively. The thickness of h-BN was set as 100 nm, and the working wavenumber was set as 1001 points to scan parameters in the range of 1–1800 cm^−1^. Figure 8 shows the electric field distributions of the structure in 1416.81 cm^−1^ and 1611.11 cm^−1^ incident wavenumbers. In Figure 8a,c, the incident light travels in the structure along the propagation direction y for 1416.81 cm^−1^, which illustrates some energy loss and a larger propagation length in this wavenumber. In Figure 8b,d, corresponding to the 1611.11 cm^−1^ incident wavenumber, the incident light cannot travel through the structure in the y direction. The findings show that there are more energy losses and a smaller propagation length at 1611.11 cm^−1^. The above results prove the different propagation lengths at different incident wavenumbers. The propagation length in the 1416.81 cm^−1^ incident wavenumber is obviously larger than that of 1611.11 cm^−1^, as shown in Figure 7. Thus, the above-mentioned theoretical analysis is validated.

**Figure 9:**
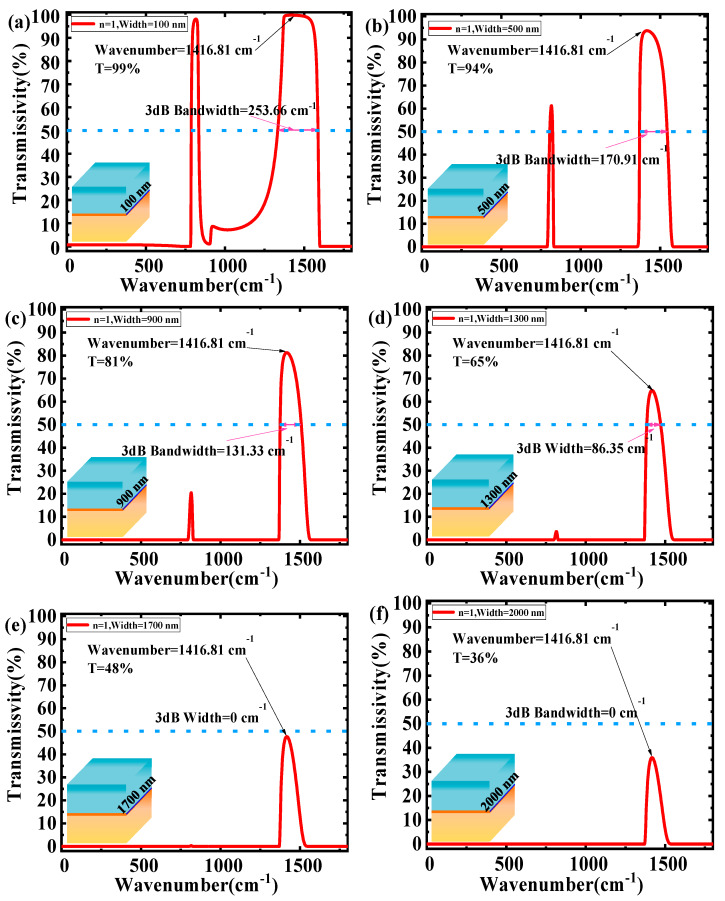
Transmissivity as a function of wavenumber in different structure width: (**a**) ε_1_ = 1, ε_2_ = 3.9, d = 100 nm and width =100 nm; (**b**) ε_1_ = 1, ε_2_ = 3.9, d = 100 nm and width = 500 nm; (**c**) ε_1_ = 1, ε_2_ = 3.9, d = 100 nm and width = 900 nm; (**d**) ε_1_ = 1, ε_2_ = 3.9, d = 100 nm and width = 1300 nm; (**e**) ε_1_ = 1, ε_2_ = 3.9, d = 100 nm and width = 1700 nm; and (**f**) ε_1_ = 1, ε_2_ = 3.9, d = 100 nm and width = 2000 nm.

After determining the values of ε_1_, ε_2_ and d, the transmissivity and 3dB bandwidth of the type-II band were calculated for different structure widths using Equation (3), and shown in Figure 9. With the increase of the structure width, the maximum transmissivity and the 3dB bandwidth obviously decrease. This is mainly attributed to the longer structure width, which causes more loss, reduces the light energy transmission and finally leads to the decrease of the maximum transmissivity and the 3dB bandwidth. 

At the 100 nm structure width, Figure 9a shows the highest transmissivity, whose value reaches ~99%. Its 3dB bandwidth is ~253.66 cm^−1^, which is suitable for the broadband filters design. With the increase of the structure width, energy loss is larger, which reduces the transmissivity more, leading to a narrower 3dB bandwidth. At the 1300 nm structure width, Figure 9d shows the narrowest 3dB bandwidth, which reaches ~86.35 cm^−1^ and can be adopted for the design of a narrow band filter. When the structure width reaches or exceeds 1700 nm, the 3dB bandwidth is equal to 0, and the structure becomes unsuitable for the design of a filter. The comparing results showed that the excellent filtering characteristics in Figure 9a are caused by the small structure width and a large excitation for h-BN in two Reststrahlen (RS) bands. For other structure widths, a longer structure width increases the transmission energy losses and weakens the stronger excitation in two RS bands.

## 4. Conclusions

In this paper, the filtering characteristics of the phonon polaritons waves in a dielectric-hexagonal Boron nitride (h-BN)-dielectric structure have been studied. The results show that improved filtering characteristics with a propagation length from 0.0028 μm to 1.9756 μm at different incident wavenumbers will be achieved, if the relative permittivity of the materials above and below h-BN is smaller and the h-BN is thicker (ε_1_ = 1 (air), ε_2_ = 3.9 (SiO_2_), d = 100 nm). The theoretical analysis was verified by simulation using the COMSOL tool. The transmissivity and the 3dB bandwidth of the dielectric-h-BN-dielectric structure with different width was calculated. A maximum transmissivity of ~99% has been achieved in a 100 nm structure width, and the minimum 3dB bandwidth reached ~86.35 cm^−1^ at 1300 nm structure width. When the structure width meets or exceeds 1700 nm, the 3dB bandwidth is equal to 0, which shows that the 1700 nm structure width is the limit for the filter application. These results suggest that the dielectric-h-BN-dielectric structure is promising to design a filter with nanostructures for real applications.
